# Elucidating a genomic signature associated with behavioral and executive function after moderate to severe pediatric TBI: a systems biology informed approach

**DOI:** 10.3389/fsysb.2024.1293265

**Published:** 2024-04-25

**Authors:** Brad G. Kurowski, Amery Treble-Barna, Valentina Pilipenko, Lisa J. Martin, Anil G. Jegga, Aimee E. Miley, Nanhua Zhang, Anthony Fabio, Ranjit S. Chima, Anna-Lynne R. Adlam, Kenneth Kaufman, Michael J. Bell, Sue R. Beers, Stephen R. Wisniewski, Shari L. Wade, TBI Genetics and Environment Study Team

**Affiliations:** ^1^ Division of Pediatric Rehabilitation Medicine, Cincinnati Children’s Hospital Medical Center, Cincinnati, OH, United States; ^2^ Department of Pediatrics, University of Cincinnati College of Medicine, Cincinnati, OH, United States; ^3^ Department of Neurology and Rehabilitation Medicine, University of Cincinnati College of Medicine, Cincinnati, OH, United States; ^4^ Department of Physical Medicine and Rehabilitation, University of Pittsburgh, Pittsburgh, PA, United States; ^5^ Division of Human Genetics, Cincinnati Children’s Hospital Medical Center, Cincinnati, OH, United States; ^6^ Division of Biomedical Informatics, Cincinnati Children’s Hospital Medical Center, Cincinnati, OH, United States; ^7^ Division of Behavioral Medicine and Clinical Psychology, Cincinnati Children’s Hospital Medical Center, Cincinnati, OH, United States; ^8^ Division of Biostatistics and Epidemiology, Cincinnati Children’s Hospital Medical Center, Cincinnati, OH, United States; ^9^ Department of Epidemiology, Epidemiology Data Center, University of Pittsburgh, Pittsburgh, PA, United States; ^10^ Division of Critical Care Medicine, Cincinnati Children’s Hospital Medical Center, Cincinnati, OH, United States; ^11^ Department of Psychology, Faculty of Health and Life Sciences, University of Exeter, Exeter, United Kingdom; ^12^ Center for Autoimmune Genomics and Etiology, Cincinnati Hospital Medical Center, Cincinnati, OH, United States; ^13^ Cincinnati Veterans Affairs Medical Center, Cincinnati, OH, United States; ^14^ Department of Critical Care Medicine, Children’s National Hospital, Washington, DC, United States; ^15^ Department of Psychiatry and Neurological Surgery, University of Pittsburgh School of Medicine, Pittsburgh, PA, United States; ^16^ Department of Psychology, University of Cincinnati, Cincinnati, OH, United States

**Keywords:** traumatic brain injury, genomics, systems biology, pediatrics, long-term, behavior, executive function

## Abstract

**Introduction:** There is significant unexplained variability in behavioral and executive functioning after pediatric traumatic brain injury (TBI). Prior research indicates that there are likely genetic contributions; however, current research is limited. The purpose of this study is to use a systems biology informed approach to characterize the genomic signature related to behavioral and executive functioning ∼12 months after moderate through severe TBI in children.

**Methods:** Participants were from two prospective cohorts of children with severe TBI (Cohort #1) and moderate-severe TBI and an orthopedic injury (OI) group (Cohort #2). Participants included 196 children (*n* = 72 and *n* = 124 total from each respective cohort), ranging in age between 0–17 years at the time of injury. In total, 86 children had severe TBI, 49 had moderate TBI, and 61 had an OI. Global behavioral functioning assessed via the Child Behavior Checklist and executive function assessed via the Behavioral Rating Inventory of Executive Function at ∼ 12 months post injury served as outcomes. To test for a genomic signature, we compared the number of nominally significant (*p* < 0.05) polymorphisms associated with the outcomes in our systems biology identified genes to a set 10,000 permutations using control genes (e.g., not implicated by systems biology). We used the ToppFun application from Toppgene Suite to identify enriched biologic processes likely to be associated with behavioral and executive function outcomes.

**Results:** At 12 months post injury, injury type (TBI vs OI) by polymorphism interaction was significantly enriched in systems biology selected genes for behavioral and executive function outcomes, suggesting these genes form a genomic signature. Effect sizes of the associations from our genes of interest ranged from .2–.5 for the top 5% of variants. Systems biology analysis of the variants associated with the top 5% effect sizes indicated enrichment in several specific biologic processes and systems.

**Discussion:** Findings indicate that a genomic signature may explain heterogeneity of behavioral and executive outcomes after moderate and severe TBI. This work provides the foundation for constructing genomic signatures and integrating systems biology and genetic information into future recovery, prognostic, and treatment algorithms.

## Introduction

Traumatic brain injury (TBI) is a leading cause of morbidity and mortality in children. Recovery is the result of a multitude of factors including injury-related, individual, social-environmental, medical, behavioral, therapeutic (e.g., physical, occupational, and speech therapy), and other factors as well as interactions among factors. (Abecasis et al., 2001; Kenzie et al., 2017; Petranovich et al., 2020; Kenzie et al., 2022; Kurowski et al., 2022). However, why outcomes differ so markedly remains unexplained and a definitive model of recovery is lacking. Emerging research suggests that genetics likely influence recovery; yet, there continue to be critical gaps in understanding the nature of these associations and which genes/gene pathways are most salient. (Reddi et al., 2022). A better understanding of the influence of genetics on recovery could help to elucidate unexplained heterogeneity and lead to individualized prognosis and management of individuals at risk for prolonged neurocognitive and behavioral problems who may require closer monitoring and more aggressive intervention.

Genetics are believed to influence brain injury recovery. Based on human and animal studies, genes involved in neurotransmitter, inflammatory, neurogenesis, and neural repair signaling, among other pathways, have demonstrated associations with outcomes after traumatic brain injury. (Kurowski et al., 2019; Treble-Barna et al., 2020; Cortes and Pera, 2021). However, most prior studies have taken narrow approaches to evaluating a single or a few genetic variants with an array of outcomes, including behavior, cognition, mortality, global functioning, and post-traumatic seizures. (Kurowski et al., 2017a; Kurowski et al., 2019). Effect sizes of these candidate approaches are modest at best and this prior research indicates that it is unlikely that a single gene or genetic variant will explain most outcomes. Highly varied TBI outcomes may be analogous to complex traits for which the effects of multiple genes or variants in concert with environmental influences will explain recovery more fully than narrow candidate gene approaches. (Goddard et al., 2016). Systems biology approaches represent a potential way to understand the complex relationships among multiple genes and outcomes. Systems biology methods represent a balance between breaking a system down into smaller parts (reductionism) and understanding how the parts work together to function as a whole (synthesis). (Hillmer, 2015). Additionally, prior TBI work using a systems biology approach suggests that a genomic signature involving multiple genes associated with brain injury recovery-related processes might better explain the heterogeneity in recovery after TBI than narrow or candidate gene approaches. (Kurowski et al., 2019).

The current multi-cohort, international project described in this manuscript uses a system biology and genomic approach, combined with gene-set enrichment methods to elucidate whether brain injury recovery genetic variants are associated with behavioral and executive function outcomes after moderate and severe pediatric TBI. We hypothesized that a genomic signature, characterized by genes linked to biologic processes critical to TBI recovery, would be more likely to be associated with neurobehavioral outcomes than a set of genes not linked to such processes. To ensure we were capturing associations specific to the brain injury, we also tested whether these genomic associations differed between children with TBI and an orthopedic injury (OI) control group of children. This project is a critical first step toward understanding whether a system of genes enriched with biologic processes important to brain injury recovery can be used to explain heterogeneity in neurobehavioral outcomes after moderate and severe pediatric TBI. Combining system biology methods, genetic association, and gene-set approaches allows for insights to be drawn from smaller sample sizes and represents an approach that is likely able to characterize the contributions of multiple genes in biologic systems important to TBI recovery, a systems-biology informed genomic signature of recovery.

## Materials and methods

Design: The present study combines participants from two prospective cohorts of participants with pediatric TBI or OI who were approached after participation in one of two larger TBI cohorts to consent to provide DNA for genetic analysis. The first cohort was enrolled from the larger Ohio Head Injury Outcomes (OHIO) study, which included children aged 3–7 years hospitalized following moderate to severe TBI or IU recruited from three tertiary care children’s hospitals and one tertiary care general hospital in Ohio. (Taylor et al., 2001; Stancin et al., 2002; Taylor et al., 2002; Wade et al., 2002; Yeates et al., 2002; Schwartz et al., 2003; Kurowski et al., 2016; Kurowski et al., 2017b; Kurowski et al., 2019; Treble-Barna et al., 2020; Rempe et al., 2022; Treble-Barna et al., 2022; Treble-Barna et al., 2023). The second cohort was enrolled from the larger Approaches and Decisions in Pediatric TBI (ADAPT) trial, an international comparative effectiveness trial of ICU interventions in children aged 0 to <18 years with severe TBI. (Bell et al., 2022; Kochanek et al., 2022). Table 1 outlines inclusion criteria for the OHIO and ADAPT studies.

**TABLE 1:** Inclusion criteria for cohorts from which participants were recruited.

	OHIO cohort	ADAPT cohort
Participant characteristics	3–7 years at time of injury, no evidence of child abuse as the cause of the injury, no history of prior TBI, documented neurological problems, or developmental delays pre-injury	Ages 0 to <18 years at time of injury and sustained a severe TBI. Children that sustained a less severe injury (GCS >8) or did not survive the injury were excluded
Hospitalization characteristics	Overnight stay in hospital	Admitted to intensive care unit with intracranial pressure monitoring
TBI severity definition	Glasgow Coma Scale (GCS) score and clinically obtained neuroimaging findings. Severe TBI was defined as a GCS score ≤8. Moderate TBI was defined as a GCS score of 9–12 with or without abnormal neuroimaging (moderate TBI) or a higher GCS score with abnormal neuroimaging as defined by an intracranial or parenchymal injury or depressed skull fracture	Glasgow Coma Scale (GCS) score ≤8
Non-TBI group	Orthopedic injury group sustained a bone fracture (not including skull fractures), had an overnight hospitalization, and did not exhibit alterations in consciousness or other signs or symptoms of head trauma or brain injury	None
Geographic location	United States, specifically Ohio	United states, multiple states, and international
Language	English primary language	Primarily English, but mixed

**FIGURE 1:**
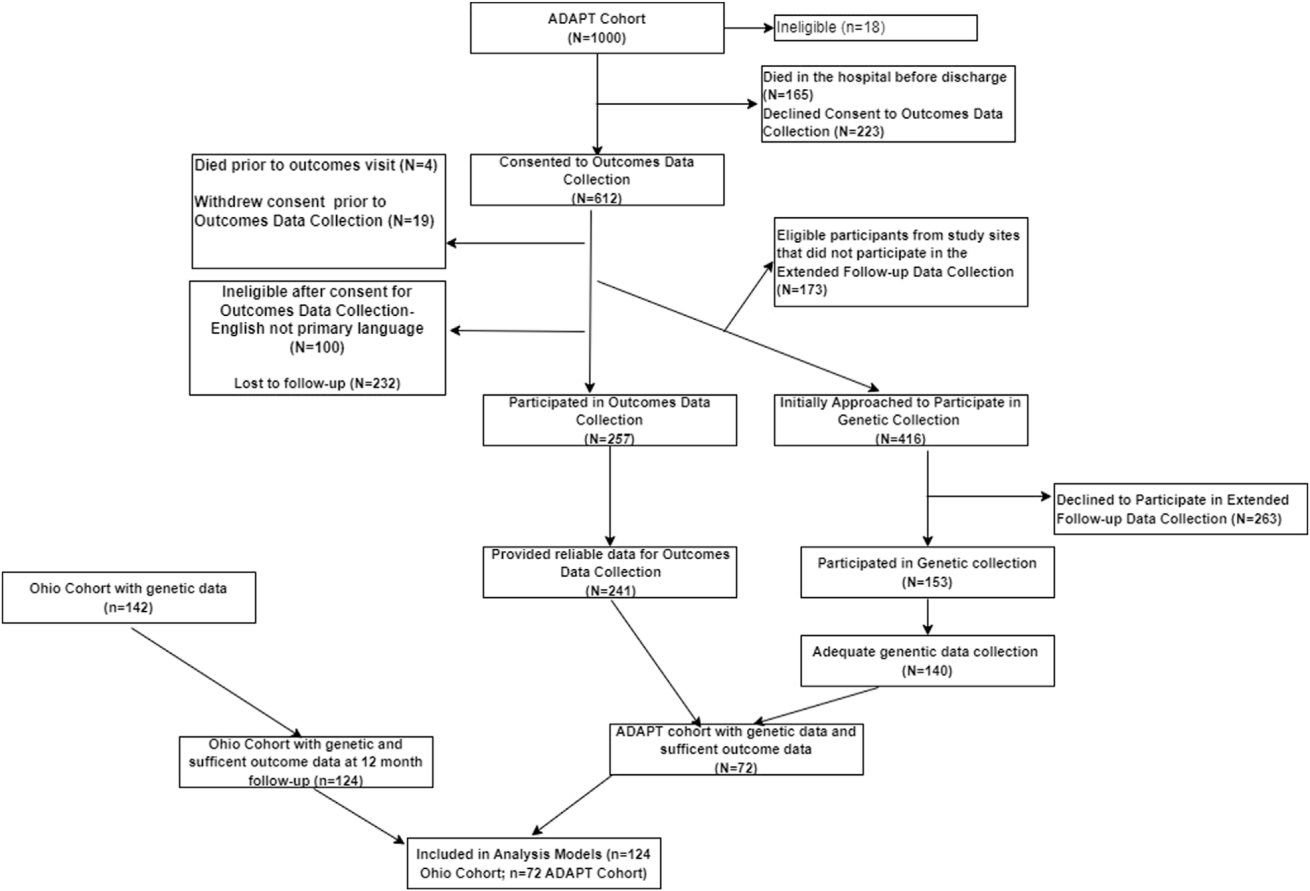
Flow chart for participants included in the analysis. The flow chart characterizing the inclusion of participants in analysis from the ADAPT and OHIO Cohorts.

Participants: Inclusion criteria for the OHIO study were overnight hospitalization for traumatic injury (TBI or OI), age between 3 and 7 years at time of injury, no evidence of child abuse as the cause of the injury, no history of prior TBI, documented neurological problems, or developmental delays pre-injury, and English as the primary language spoken in the home. TBI severity was determined using the lowest post-resuscitation Glasgow Coma Scale (GCS) (Teasdale and Jennett, 1974) score and clinically obtained neuroimaging findings. Severe TBI was defined as a GCS score ≤8. Moderate TBI was defined as a GCS score of 9–12 with or without abnormal neuroimaging (moderate TBI) or a higher GCS score with abnormal neuroimaging as defined by an intracranial or parenchymal injury or depressed skull fracture. Children in the OI group sustained a bone fracture (not including skull fractures), had an overnight hospitalization, and did not exhibit alterations in consciousness or other signs or symptoms of head trauma or brain injury. Participants included in the ADAPT trial consisted of youth who were ages 0 to <18 years at time of injury and sustained a severe TBI as defined by a Glasgow Coma Scale (GCS) score ≤8; who were admitted to the pediatric intensive care unit (PICU) and underwent placement of an intracranial pressure (ICP) monitor. (Bell et al., 2022; Kochanek et al., 2022). Children that sustained a less severe injury (GCS >8) or did not survive the injury prior to consent were excluded. Attempts were made to contact all participants from each cohort to consent for collection of saliva samples and completion of outcomes assessment. All study activities were completed in accordance with the Helsinki Declaration, approved by the institutional review boards at participating sites, and parental/guardian consent was obtained in accordance with the Declaration of Helsinki. Figure 1 illustrates the participants included in the present analysis: Total n = 196, OI n = 61, Moderate TBI *n* = 49, Severe TBI *n* = 86. Table 2 compares the characteristics of participants by injury group. Models were developed using these injury groups consistent with prior research examining outcomes after pediatric TBI. (Kurowski et al., 2016; Treble-Barna et al., 2017; Kurowski et al., 2019; Treble-Barna et al., 2020; Treble-Barna et al., 2022; Treble-Barna et al., 2023).

**TABLE 2:** Participant characteristics by injury type.

Characteristic	Overall, N = 196^a^	OI, N = 61^a^	Moderate, N = 49^a^	Severe, N = 86^a^	*p*-value^b^
Sex					0.4
Female	103 (53%)	30 (49%)	23 (47%)	50 (58%)	
Male	93 (47%)	31 (51%)	26 (53%)	36 (42%)	
Age at injury, years	5.7 (4.3, 7.2)	5.2 (4.2, 6.0)	5.2 (4.1, 6.1)	10.3 (4.7, 13.9)	<0.001
Time since injury, years	1.08 (1.0, 1.2)	1.11 (1.1, 1.2)	1.16 (1.1, 1.2)	1.04 (1.0, 1.1)	<0.001
Ancestral populations					0.2
Ad Mixed American	11 (5.6%)	1 (1.6%)	2 (4.1%)	8 (9.3%)	
African	34 (17%)	9 (15%)	11 (22%)	14 (16%)	
East Asian	2 (1.0%)	0 (0%)	0 (0%)	2 (2.3%)	
European	146 (74%)	51 (84%)	36 (73%)	59 (69%)	
South Asian	3 (1.5%)	0 (0%)	0 (0%)	3 (3.5%)	
Ethnicity					0.7
Hispanic or Latino	6 (3.1%)	3 (4.9%)	2 (4.1%)	1 (1.2%)	
Non-Hispanic or Latino	165 (84%)	58 (95%)	47 (96%)	60 (70%)	
Missing	25 (13%)	0 (0%)	0 (0%)	25 (29%)	
CBCL	50 (41, 59)	41 (38, 50)	52 (42, 57)	56 (47, 65)	<0.001
Missing	4	0	0	4	
BRIEF	53 (43, 63)	45 (41, 52)	55 (42, 61)	60 (50, 70)	<0.001
Missing	3	0	0	3	

^a^ n (%); Median (IQR).

^b^ Pearson’s Chi-squared test; Kruskal–Wallis rank sum test; Fisher’s exact test.

Demographic and descriptive data of participants in the analysis. CBCL, child behavior checklist; BRIEF, behavior rating inventory of executive function.

*Outcome Measures: Executive Functioning:* The Behavior Rating Inventory of Executive Function (BRIEF) (Gioia et al., 2002; Vriezen and Pigott, 2002; Gioia and Isquith, 2004; Karunanayaka et al., 2007; Power et al., 2007; Wozniak et al., 2007; Muscara et al., 2008a; Muscara et al., 2008b; Conklin et al., 2008; Merkley et al., 2008; Sesma et al., 2008; Walz et al., 2008; Chevignard et al., 2009; Donders et al., 2010) preschool (2 - <6 years), school age (6 - < 18 years) and adult (>18 years) versions were used to assess executive functioning. The BRIEF has been well validated in pediatric TBI. (Gioia et al., 2000; Gioia and Isquith, 2004; BRIEF Behavior Rating Inventory of Executive Function, 2021). The global executive composite (GEC) T score served as the measure of executive function behaviors, with higher scores indicating greater executive dysfunction. *Behavior:* The Child Behavior Checklist (CBCL) (Fletcher et al., 1990; Ewing-Cobbs et al., 2004; Beebe et al., 2007; Chapman et al., 2010) was used as the primary behavioral measure in the study. The CBCL–infant/toddler was used for children ages 1.5–5 years, and the CBCL - child was used for children >5 years. The CBCL total problems T score was used as the measure of behavior problems to provide a comprehensive assessment of parent-reported internalizing and externalizing behavior problems across domains, with higher scores indicating more behavior problems. Both measures were proxy-reports completed by parent/guardian. Measures were administered by research staff for each cohort at ∼ 1 year post injury (Mean = 1.08 years, IQR: 1.0–1.3). Pearson correlation analysis between measures indicated high correlation (r = .81).

*Genetic Sample Collection:* Collection of saliva samples occurred via mail or through an additional in-person visit. Samples were checked to ensure adequate quality of DNA purification. If problems were noted with the purification, attempts to repeat collection of the sample were undertaken. The Oragene (DNA Genotek, Ottawa, Ontario, Canada) DNA Self-collection kit was used. (Nunes et al., 2012). DNA was extracted using the manufacturer’s recommended procedure for Oragene samples. DNA from saliva was extracted on a Promega Maxwell 16 nucleic acid extractor (Promega Corporation, Madison, WI) according to manufacturer’s instructions. Extraction was performed via magnetic bead-based protocol. Following extraction, genomic DNA was assayed on a Lunatic nucleic acid quantification System (Unchained Labs, Pleasanton, CA) for quality and quantity. Completion of DNA extraction and genotyping was performed using the genetics core at CCHMC. Genotyping for OHIO cohort was done using the HumanExome v1.1 Bead Chip (Illumina, San Diego, CA) and for ADAPT cohort we used the Omni 2.5 Omni2.5-8v1.5 chip (Illumina, San Diego, CA) in accordance with manufacturer’s protocols. (Fan et al., 2003; SNP Genotyping and Copy Number Analysis, 2023).

**FIGURE 2:**
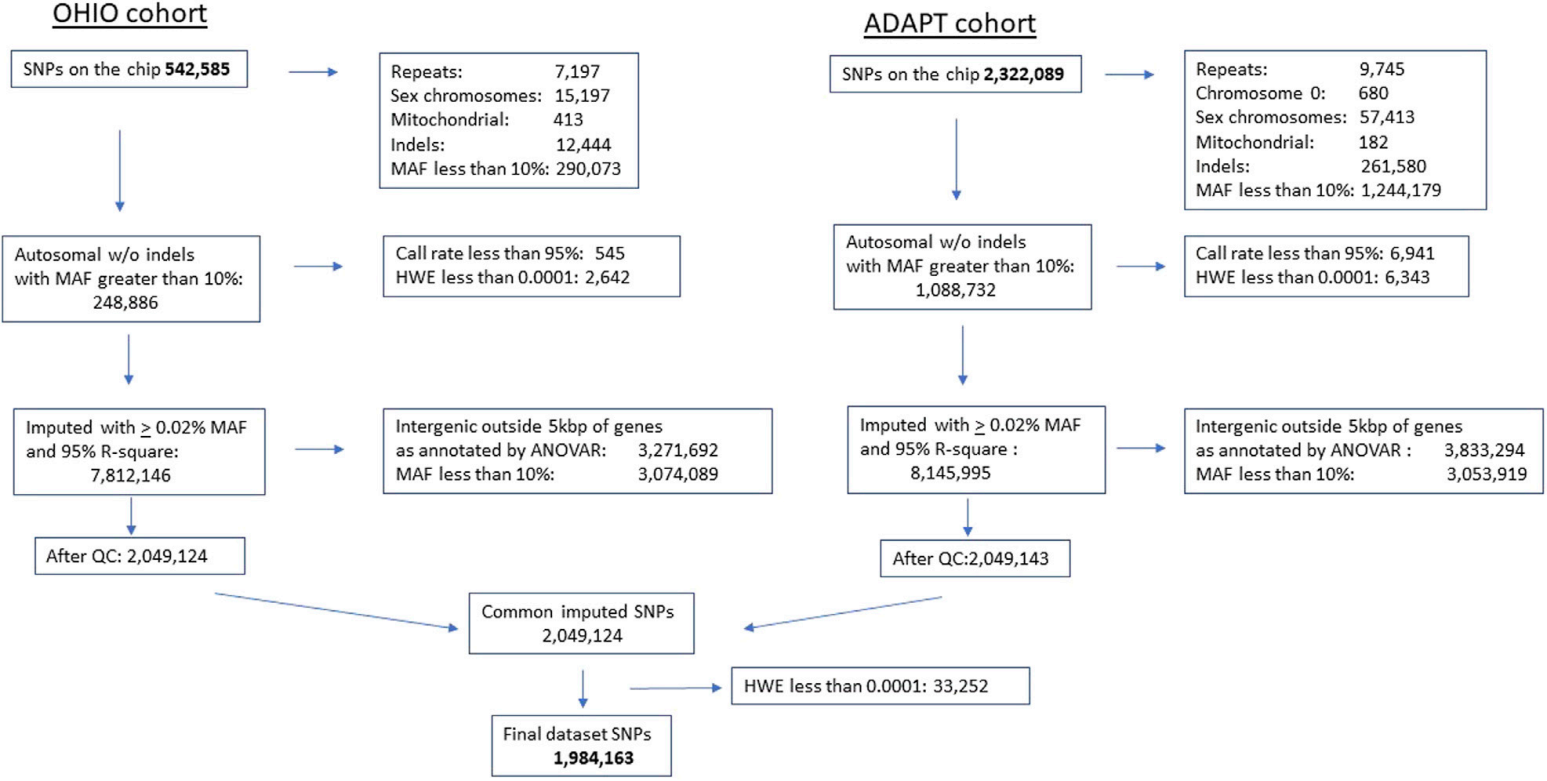
Single nucleotide polymorphism chip processing flow. Single nucleotide polymorphism gene chip processing flow diagram. Final dataset included 1,984,163 single nucleotide polymorphism after quality control, processing, and imputation. SNP, single nucleotide polymorphism, MAF, minor allele frequency, HWE, Hardy Weinberg Equilibrium violated at *p* < 0.0001, QC, quality control, ANOVAR, Analysis of Variance.

*Data quality control and genetic variant imputation and annotation:* Prior to conducting any genetic analysis, we examined the genetic data for rare and poor quality genetic variants. To be included, we required a minor allele frequency of at least 10%, ≥95% at a SNP level and 90% at participant level to be assigned a genotype for each variant. Variants were excluded if Hardy-Weinberg equilibrium was violated (*p* < 10^–4^) to reduce chance for aberrant associations. (Turner et al., 2011). Testing for cryptic relatedness was done using graphical representation of relationship (GRR). (Abecasis et al., 2001). Prior to statistical analysis, we also examined the distribution and quality of the outcome data on the CBCL and GEC. *Imputation and Annotation*: Variants from both chips that passed quality control were submitted for imputation using the TOPMed Imputation Server on the NIH BioData Catalyst using the TopMed R2 reference panel. (TOPMed Imputation ServerFree Next-Generation Genotype Imputation Service, 2024). A total of 1,088,732 variants were submitted for imputation from the Omni2.5-8v1.5 and 248,886 variants from the HumanExome v1.1Bead Chip. In the imputed data, we removed variants with an Rsq <0.95 and a minor allele frequency less than 10%. Our final dataset included 1,984,163 imputed variants, which corresponds to 2,708 genes. Annovar software (Wang et al., 2010) was used to annotate genetic variants. We excluded intergenic variants, defined as variants with more than 5,000 base-pairs from the closest genes. See Figure 2 for single nucleotide polymorphism processing flow chart.

System biology pathways and processes to identify case and control gene-sets: Information from a literature review was used to identify known TBI-associated genes. (Kurowski et al., 2017a). In this prior work, 18 genes were identified that previously showed an association with neurocognitive and behavioral outcomes after TBI: (Kurowski et al., 2017a): angiotensin I converting enzyme (ACE), adenosine A1 receptor (ADORA1), Ankyrin repeat and kinase domain contacting 1 (ANKK1), apolipoprotein E (APOE), BCL2, apoptosis regulator (BCL2), brain derived neurotrophic factor (BDNF), BMX non-receptor tyrosine kinase (BMX), catechol-O-methyltransferase (COMT), dopamine beta-hydroxylase (DBH), fatty acid amide hydrolase (FAAH), glutamate decarboxylase 1 (GAD1), glutamate ionotropic receptor NMDA type subunit 2A (GRIN2A), monoamine oxidase A (MAOA), NADH dehydrogenase, subunit 1 (MT-ND1), NADH dehydrogenase, subunit 3 (MT-ND3), neuroglobin (NGB), solute carrier family 6 member 4 (SLC6A4), WW and C2 domain containing 1 (WWC1). These 18 genes represented our known or “training” gene list. The training gene list was then evaluated using a systems biology platform, ToppGene Suite. (Chen et al., 2007). ToppGene Suite is a comprehensive platform used for gene set enrichment analyses and machine learning-based candidate gene ranking. (Chen et al., 2009). The ToppGene methods are described in detail elsewhere and we refer the reader to these methods (https://bmcbioinformatics.biomedcentral.com/articles/10.1186/1471-2105-8-392#Sec15). (Chen et al., 2007) ToppGene was used to identify brain injury recovery related biologic processes and pathways using the training set of genes, and then additional genes, i.e., “case” genes (exclusive of the training set), likely to be highly related to brain injury recovery related biologic processes were identified. The case genes were ranked based on their functional similarity to the training set using the ToppGene application with default parameters. (Chen et al., 2007). The functional similarity between the training and case genes was computed using a variety of gene annotations: pathways, biological processes, phenotype, literature, protein interactions, and co-expression as described previously. (Chen et al., 2007). *Control gene set identification*: To identify a control set of genes, i.e., genes unlikely related to brain injury recovery, we used results from the ToppGene Suite analysis described above to identify genes *not* enriched in biologic processes and pathways associated with brain injury recovery.

*Analytic methods:* We combined systems biology and genetic association testing methodologies to evaluate whether genes/variants associated with biologic processes critical to TBI recovery (i.e., training plus “case” gene set) were more likely to demonstrate associations (i.e., enrichment) with behavioral and executive function outcomes compared to genes/variants not associated with these biologic pathways (i.e., control gene set).

*Genetic ancestry:* Because continental ancestry is a potential confounder for genetic association studies, we used a set of Ancestry Informative Markers (AIMs) to estimate continental ancestry using principal components (PC) analysis (Patterson et al., 2006). The 1000G data were used as an anchor for continental ancestry. (1000 Genomes Project Consortium et al., 2015). Ancestral populations were divided into Ad Mixed American, African, East Asian, European, and South Asian following 1000G super population classification. Using ancestral population categories allowed for consistent comparison across participants from different countries. We evaluated the scree plot to empirically determine the number of PCs, and found one PC was sufficient for representing genetic similarity (PC1).

*Demographic and outcome measure comparison between groups*: Sex, genetic similarity (PC1), age at injury, time since injury at follow-up, CBCL, and GEC scores were compared between TBI and OI groups and the two cohorts using two-sample t-tests for continuous variables or Chi-squared tests for categorical variables.

*Model development:* Prior to the genetic analyses, we used multivariable regression to identify potential covariates. The primary goal of the non-genetic models was to use covariates to account for non-genetic variation and then evaluate whether genetic factors account for substantial additional variation in outcomes. Initial non-genetic models developed for the CBCL and GEC outcomes incorporated demographics (age at injury, sex, genetic similarity (PC1), an interaction term between age at injury and sex, and injury group (3-levels moderate TBI, severe TBI, or OI). Inclusion of the interaction term of age at injury and sex accounted for significant variance in outcomes; therefore, age at injury, sex, and their interaction were carried forward in subsequent analyses. PC1 from the genetic ancestry analyses was included in all models. To ensure cohorts (ADAPT *versus* Ohio) from which participants were recruited did not add to variation in the model beyond the injury group, we investigated how inclusion of the cohort term affected the models. See Supplementary Table S1 for comparison of participants across injury group and cohorts (ADAPT and Ohio) from which they were recruited. Our analysis indicated that cohort (ADAPT *versus* Ohio) did not account for substantial additional variation beyond the injury group variable in models based on R-squared change (R-square change <0.2% and *p* > 0.5); therefore, only the injury group term was included in final models. Genetic models included a genetic variant term, the interaction term between group and genetic variant, in addition to variables in the non-genetic model. To test for genetic variant association, we used linear models to test for association of each variant as well as variant x injury group interaction (to test whether a SNP’s association with outcome differed by injury group). In all association tests, we used an additive genetic model where major homozygotes were coded as 0, heterozygotes as 1, and minor homozygotes as 2. To provide effect size estimates, we standardized all continuous variables (M = 0, SD = 1) prior to analyses and obtained parameter estimates based on the final model for each dependent variable. The resulting coefficients are akin to standardized regression coefficients for continuous predictors and to standardized mean differences (e.g., Cohen’s *d*) for categorical variables. Because standardized regression coefficients can be scaled to correlations (Cohen, 1988), we used conventional definitions of effect size to characterize the magnitude of standardized parameter estimates for continuous predictors (i.e., 0.1 is small, 0.3 is medium, and 0.5 is large). Likewise, we used conventional definitions of effect size for mean differences to characterize the magnitude of parameter estimates for categorical predictors and any interactions involving them (i.e., 0.2 is small, 0.5 is medium, 0.8 is large). Analyses were conducted using PLINK version1.9. (Purcell et al., 2007).

Gene-set analyses were performed using R software (http://www.R-project.org) to test whether there was a greater number of genetic associations present in the case vs control set of genes. We compared the number of associations in our case set that met the *p* < 0.05 threshold to the number of associations meeting the same criteria in over 10,000 matched runs of our control set of genes. Given the large number of case genes, we used the system biology-based rankings to create subsets of the larger set of ranked genes to determine if a more restrictive list was sufficient. We created subset gene lists and the full gene list. These lists include 0% (training set only), 5% (training set plus top 5% of ranked genes), 10% (training set plus top 10% of ranked genes), etc., until all training and case genes were included. Thus, these lists are described as percentiles of case genes. We then selected sets of control genes (for each set of case genes, 10,000 control genes were selected for each set). SNPs for the control set were matched to the case gene set on the ratio of minor allele frequency (MAF) using MAF bands as follows: 10%–15%; 15%–20%: 20%–30%; 30%–50%. Using the 10,000 matched runs from the control sets, we established the 95th percentile for the number of associations expected by chance. When the number of nominal (*p* < 0.05) associations in the case genes exceeded the 95th percentile expected by chance (North et al., 2002), the case genes at these percentile epochs were considered to be enriched for genes/SNPs associated with each outcome individually. While we recognize that many of the nominally associated genes are false positive associations due to an inflated family-wise error rate, our question is whether there is enrichment of association across the overall set of variants (rather than any specific variant). Therefore, we did not correct for multiple testing among the cumulative subsets of variants as these are not mutually exclusive subgroups. The 10,000 matched control runs (for each comparison group) ensures that the *p*-value accounts for the number of variants tested. Separate analyses were performed for the behavioral and executive function outcomes.

*Precision biologic process identification based on genetic association analysis:* To identify what genes/pathways contributed the largest effects more precisely on outcomes, we explored whether genes associated with variants having the top fifth centile of effect sizes with behavioral and executive function outcomes in this analysis continued to implicate prior biologic processes identified. We used the ToppFun application from Toppgene Suite (Chen et al., 2009) to identify which biologic processes were significantly enriched and likely to be associated with behavioral and executive function outcomes. Benjamini–Hochberg (Galwey, 2023; Yang et al., 2024) correction was used for multiple testing in the gene-enrichment analysis.

## Results

Genetic data were collected from 196 participants (Table 2). The median age was 5.7 years (IQR 4.3–7.2), 103 were female. Based on genetics, 74% were European descent, 17% were African descent, 8% had other ancestry. The ADAPT cohort was older on average than the OHIO cohort. The OI group had lower (better) scores on the CBCL and GEC compared to the TBI groups.

**FIGURE 3:**
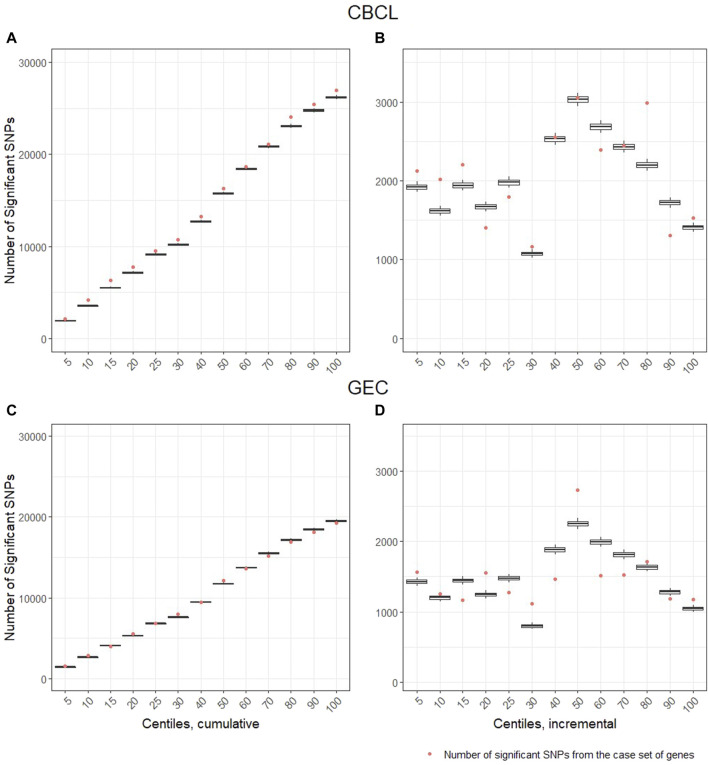
Gene-set analysis and genetic enrichment. The vertical axis is the number of nominally significant variants (*p* < .05). The horizontal axis represents the centiles based on ranked genes from the systems biology approach. The centile lists 5% (training set plus top 5% of ranked genes), 10% (training set plus top 10% of ranked genes), etc., until all (i.e., 100%) training and case genes were included, thus, these lists are described as percentiles of case genes. The dot represents the number of significant genetic variants by injury group interactions identified in the case set of genes. The box and whisker plots represent the distribution of the significant variant by injury group interactions identified in the control set of genes. When the dot is above the whisker, this pattern represents enrichment in the case set of genes. That is, it represents the 95% confidence cut-off at which there is a greater number of variants by injury group interactions in the case set of genes than expected when compared to the control set of genes. Panels **(A, C)**, represent cumulative number of genetic variants by injury group interactions and panels **(B, D)**, represent the within centile relationship. Panels **(A, B)** are the Child Behavior Checklist (CBCL) outcome and panels **(C, D)** are the Behavioral Rating Executive Function (BRIEF) Global Executive Composite (GEC) outcome.

*Gene-set analysis results*: For the CBCL total at 12 months post injury, we found significant enrichment for the injury group by variant interaction across multiple deciles of ranked genes. The number of significant brain injury recovery ranked variants associated with total behavior problems was greater than expected by chance at the 95 percentile compared to the control set of genes (Figure 3). When evaluating the cumulative effect of these ranked genes, we find that across all deciles (Figure 3A), the number of nominally significant SNPs in genes within the ranking, represented as dots in Figure 3A, are higher than the control set. The incremental effect (Figure 3B) supports enrichment within the fifth, 10th, 15th, 30th, 80th, and 100th centiles. These results suggest that the genomic signature, i.e., aggregate signal from the ranked genes, accounted for substantial variability in total behavior problems and significantly by injury group. For executive function behaviors (GEC) at 12 months post injury, cumulative enrichment effects for the injury group by variant interaction for our ranked genes were less robust than the CBCL model (Figure 3C). The incremental effect supports enrichment at the fifth, 20th, 30th, 50th, and 100th centile (Figure 3D).

To further characterize the relationship of genes with outcomes, we evaluated the standardized effect sizes of the variants in our case set of genes (Table 3). Most variants had small effect sizes. For the interaction effect, there were a modest number of variants with small to medium effect sizes. When considering the main effect in the TBI group, the effect size distribution was also small to medium, see Table 3. Of note the CBCL outcome was associated with more variants in the medium effect size range than the GEC. These small and medium effect size magnitudes are consistent with the concept that the overall genetic influence on outcomes after TBI is driven by the combined contribution of multiple genes.

**TABLE 3:** Variant effect size magnitude range for interaction and TBI only models and the number of associated genes.

Outcome	Effect size*	SNPs_interaction	Genes interaction	SNPs_TBI	Genes TBI
GEC	0.2	36,121	1,012	9,702	621
	0.3	5,865	437	237	54
	0.4	406	92	0	0
	0.5	4	3	0	0
	<0.2	361,428	1,164	393,885	2,033
CBCL	0.2	47,026	977	10,439	778
	0.3	9,921	612	276	76
	0.4	1,049	154	5	2
	0.5	84	20	0	0
	<0.2	345,744	945	393,104	1,852
	Total	403,824	2,708	403,824	2,708

*Effect size to characterize the magnitude of standardized parameter estimates for continuous predictors and main effects is as follows: 0.1 is small, 0.3 is medium, and 0.5 is large. Likewise, conventional definitions of effect size for mean differences to characterize the magnitude of parameter estimates for categorical predictors and any interactions involving them is as follows: 0.2 is small, 0.5 is medium, 0.8 is large. SNPs_interaction = number of variants associated with each effect size magnitude for the interaction model; Genes interaction = number of genes associated with variants in the interaction model; SNPs_TBI, number of variants associated with each effect size magnitude for the TBI, only model; Genes TBI, number of genes associated with the variants in TBI, only model; GEC, global executive composite; CBCL , child behavior checklist; TBI, traumatic brain injury; SNP, single nucleotide polymorphism.

**FIGURE 4:**
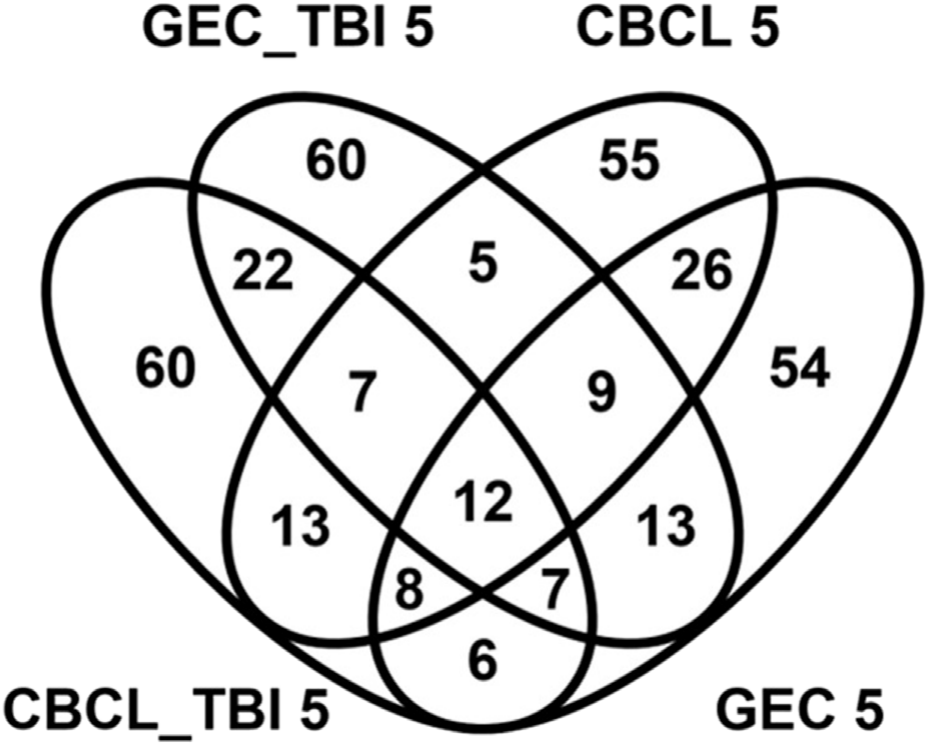
Venn diagram demonstrating overlap genes associated with genetic variants in the top 5% for effect size magnitude. The venn diagram demonstrates the magnitude of overlap among genes associated with genetic variants in the top 5% effect sizes for the genetic variant by group interaction (CBCL 5 and GEC 5) models and main effect models of variants within the TBI only (GEC_TB 5I and CBCL TBI_5) groups for both the behavioral (CBCL) and executive function (GEC) outcomes. This diagram indicates there is modest overlap in the genes across these models. 12 genes were common across all the models.

**FIGURE 5:**
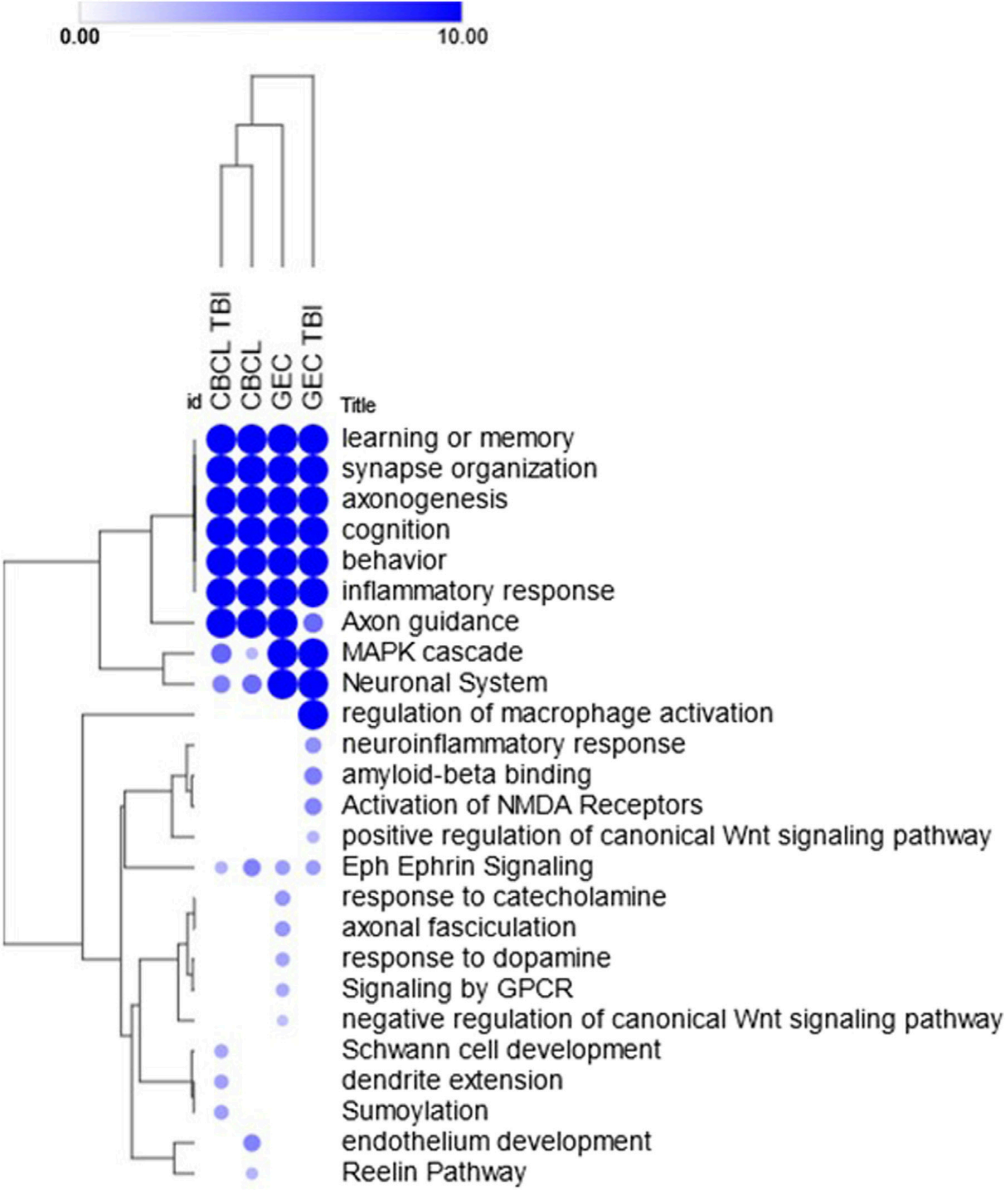
Heat map of select enriched features across the models. Heat map demonstrates the hierarchically clustered biological processes, pathways, phenotypes (rows) significantly enriched across the models using the interaction term genetic variant by injury group (CBC 5 and GEC 5) and main effect (GEC_TBI and CBC TBI_5) only TBI models (columns) for both the behavioral (CBC) and executive function (GEC) outcomes. Genetic variants demonstrated the most association with learning or memory, synapse organization, axonogenesis, cognition, behavior, and inflammatory response across all four models. Enrichments were performed using the ToppFun application of the ToppGene Suite and the heatmap was generated using the Morpheus application (https://software.broadinstitute.org/morpheus) with color intensity and size of the circles being proportional to the significance (negative log *p*-values). Complete list of enrichment results are included in the Supplementary Table S1.

*Known and novel biologic processes identified from data*: Functional enrichment analysis using the ToppFun application of the ToppGene Suite of the genes most highly associated (i.e., genes with effect sizes in the top fifth centile) with outcomes in the interaction and TBI only models indicated a high association with several expected and potentially novel biologic pathways and processes. We used the top fifth centile of the ranked genes for each outcome. A comparison of these genes across outcomes showed modest overlap, suggesting overlapping but non-identical genomic signatures for behavioral and executive function outcomes (Figure 4). However, these gene sets shared several pathways and biological processes suggesting the contribution of different genes to the same pathway or biological processes (Figure 5). Learning/memory, synapse organization, axonogenesis, behavior, and inflammatory response related biologic pathways were highly implicated as contributing to outcomes.

## Discussion

Our results demonstrate that a genomic signature, linked to *a priori* defined TBI recovery biologic systems, was associated with behavioral and executive function outcomes after pediatric TBI. This project evaluated the association of a genomic signature, measured using a genome-wide SNP chip, with behavioral outcomes in participants with moderate and severe pediatric brain injury compared to an orthopedic injury group. Consistent with our hypothesis, the genomic signature was differentially associated with outcomes after TBI. Notably, the majority of effect sizes were modest, supporting an oligogenic/polygenic genetic inheritance contributing to recovery. As such, there is likely a need to move away from single variant or even single gene-based effects in TBI-genetic investigations because single or narrow gene or variant models are unlikely to be informative without taking into account broader genomic effects. Considering the genes in aggregate, the results suggest that there are multiple biologic pathways important to TBI recovery, with patterns of gene-enrichment highlighting learning/memory, synapse organization, axonogenesis, behavior, and inflammatory response related biologic pathways. These results support the value of using a systems biology informed genomic approach to elucidate genetic determinants of TBI recovery and to provide insight into candidate biologic pathways to explore.

We found that we could detect a genomic signature associated with behavioral and executive function outcomes that differed. The genomic signature approach described here is novel. It is informed by system biology insights that identified genes associated with biologic pathways and processes important to TBI recovery and evaluated the cumulative association of many genetic variants to behavioral and executive function outcomes after moderate to severe pediatric TBI. The findings from this study build on prior work examining the influence of inflammatory polygenic risk scores on behavioral outcomes after pediatric TBI and the combined influence of several dopamine-related polymorphisms on outcomes after pediatric brain injury. (Treble-Barna et al., 2017; Treble-Barna et al., 2020). The genomic signature approach is a more genome focused and moves the field beyond single candidate variant/gene approaches and even risk score approaches that typically consider a very small number of variants in the genome. Further, the genomic signature approach described here benefits from system biology insights. Our primary genomic signature was based on the interaction between injury type and genetic variants. Using a typical single variant approach, researchers would evaluate in more detail to understand the nature of the interaction. However, in this genomic approach we are considering hundreds to thousands of variants jointly (>1 million) in the models and it is outside the scope of this analysis to deconstruct the individual interaction terms. The interaction indicates differential effects by injury type and analyses are hypothesis generating, not hypothesis testing in this case. Examining a single variant misses the larger context of the genomic signature. Indeed, we expect that for some variants the interaction may be that associations become stronger with increasing severity and others become weaker, while others may have opposite effects for severe vs non-TBI groups. Rather, the fact that the injury type by variant genomic signature is significantly enriched in our systems biology informed gene set suggests that the genomic signature is not simply capturing behavioral outcomes broadly, but rather, it is specifically capturing aspects of the heterogeneity in outcomes.

This study also provides insight into the pattern of the association of genetic factors with behavioral and executive function outcomes. Since the CBCL and GEC are known to be highly correlated (r = .81 data for this manuscript), it was expected that the pattern of genomic associations with these outcomes would be similar. However, the case genes were more consistently associated with the behavioral problems assessed by the CBCL compared to the executive function behaviors measured by the GEC. This novel observation indicates that associations of genes with different neurobehavioral outcomes are overlapping, but there are observable differences in the genes associated with different types of neurobehavioral outcomes after pediatric TBI. More broadly, this finding suggests that there may be genetic factors that could differentiate between behavioral and executive functioning as measured by the CBCL and BRIEF GEC, respectively. This observation has potential implications for considering how genetic factors might influence executive and behavioral functioning in typically developing children and other congenital and acquired conditions. (Greene et al., 2008; Gaysina, 2022).

Further, apart from leading to new insights into the biological underpinnings of outcomes after TBI, the systems biology guided approach used will encourage the development of new and innovative research on the pathways and biological processes that have been hitherto understudied in TBI outcomes and recovery. Specifically, we found that the majority of effect sizes identified were modest. This finding is consistent with findings from other GWASs of complex traits. However, it has substantial implications for TBI recovery genetic studies. To ensure that one is powered to detect modest effect sizes at the genome wide significance threshold, beyond 100,000 participants may be required. To minimize the risk of false positive associations, candidate genes/variants may be selected. However, the selection of candidates may be biased. To overcome this concern, we leveraged a systems biologic approach which considers multiple lines of evidence in determining the relationship between genes. Additionally, the systems biology approach, apart from partially addressing the limitations of the conventional gene-based approaches, can have direct implications to translational research including biomarker and drug discovery process. Combining genomic, systems biology, and detailed phenotyping has the potential to change the field and move from genetic to genomic-based approaches. Using this approach, genes harboring the largest effect sizes were associated with several shared biologic processes. The processes most strongly associated with the genomic signature were learning/memory, synapse organization, axonogenesis, behavior, and inflammatory response related biologic processes (Figure 5; Supplementary Table S2).

Consistent with prior work, the biologic processes associated with the genomic signature have also been identified as important biologic processes in TBI recovery in model organism studies. Cellular processes important to remapping of neural circuits include axonal sprouting, synaptogenesis, and synaptic pruning (i.e., synapse organization and axonogenesis). (Yu et al., 2016; Cortes and Pera, 2021). Further, injury related processes, like inflammatory response, are critical biological responses to injury that are the precursors to repair. (Marion et al., 2018; Cortes and Pera, 2021). The recovery process also invokes cellular based mechanisms important to learning and memory, including long-term potentiation and dendrite formation. (Marion et al., 2018; Cortes and Pera, 2021). This is the first pediatric TBI genomic study to create a genomic signature linked to critical brain injury recovery processes.

*Limitations:* Although one of the largest genetic studies in pediatric TBI to date, a main limitation to this study is the limited sample size; however, the significance of the approach used is the ability to perform genome wide studies for outcomes for a complex phenotype that are challenging to collect tens of thousands or more participants. The limitations, in this regard, are that we used the liberal nominal threshold for significance, so many of the variants identified may have simply been associated due to chance. However, by comparing a set of selected genes to a control set of genes, we controlled for this error. Nonetheless larger sample sizes may provide increased specificity for associations. The study also only included individuals that survived moderate and severe TBI, thus we are unable to make conclusions on the association of genetics with survival. Participants were recruited across two cohorts; therefore, there is potential for an unaccounted difference that may introduce bias; however, because models with and without a cohort specific variable did not significantly differ, it is likely that the cohort effect is nonexistent or very minimal. Further, because this study focused only on individuals that survived a moderate and severe brain injury, conclusions about whether the association of genetics with outcomes would be similar in milder TBIs, non-traumatic brain injuries or other trauma populations is unclear. Other limitations include the lack of longitudinal data, so we are unable to evaluate the association with changes in behavior and executive function over time after injury. Pre-injury measures of outcomes are unavailable; therefore, it is difficult to discern how pre-injury behavioral and executive function impacted outcome assessment at ∼12 months post injury. A large proportion of participants in the ADAPT cohort did not participate in genetic sample collection and longer-term outcome assessment; therefore, there is potential bias related to those who participated in the longer-term data collection. Participants in this study were primarily English speaking. There may also be unknown variations in acute and longer-term treatment approaches within and between the groups. In regard to polymorphism and gene selection, we selected the top 5 centile for the effect size because we felt like the number of genes to consider for enrichment analyses was optimal. The concern about a 1% threshold was that there would have been too few genes and likely driven by sporadic findings. A 10th centile was considered, but the number of genes may have been too many and would have generated more noise and been uninformative. Additionally, due to the complex nature of TBI recovery, genetic contributions are likely a combination of the small effects of many genes rather than any particular gene, therefore, we attempted to balance being too stringent and being too inclusive and creating unintended noise. We would expect that changing the number of genes considered could impact the results but did not test this formally. Despite these limitations, these findings indicate there is a high likelihood that a genomic signature of TBI can be identified, even in a diverse sample. Larger studies are needed to begin to define nuances and move toward a more individual, precision-based medicine approach.

*Future directions:* Future work will need to continue to evolve toward more polygenic and genomic approaches to understand the complex interplay among genes, associated polymorphisms, and outcomes after TBI. While prior TBI-genetic studies have noted modest effect sizes, they were still limited to a single gene or a handful of genes. Our systems biology approach demonstrates that there are many genes contributing to TBI outcomes, and these genes underpin or are enriched in certain biologic processes. Future genomic research should focus on these biological processes with the goal of potentially identifying genetic signatures of TBI recovery. This manuscript uses foundational methods that combine systems biology and genetic association methodology and provides the field with a path toward identifying genomic signatures to inform prognostication and future precision medicine approaches in pediatric TBI. Additionally, methods that combine environmental and polygenetic and genomic approaches will be needed to move the field closer toward precision prognosis and management. Epigenetic approaches should also be considered in the future along with performing analyses to understand the association of genetics with endophenotypes, like structural and functional brain imaging. (Bigos et al., 2016; Dixson et al., 2018; Greene et al., 2008; Gaysina, 2022). Studies focused on mild TBI populations are also needed. A next step in the evolution of this project would potentially be a larger scale study that includes more diverse population, the spectrum of TBI severity (mild to severe), environmental factors, and a combined omics approach that includes expression and epigenetic information in addition to genomic factors. Collecting a larger sample would be resource intensive and would require coordination and collection of common data elements (McCauley et al., 2012; Catroppa et al., 2023; Fitzgerald et al., 2023) to fully reach its potential. Collection of a diverse population regarding injury severity, demographic, and environmental factors can elucidate the heterogeneity in recovery after TBI. The current study provides a methodologic framework to use in such a larger study. Elucidating the association of genetic factors with outcomes after pediatric TBI is nascent and will require team-based science approaches to truly move the field forward.

## Conclusion

This project builds on prior work and indicates that using a genomic signature approach may help to explain heterogeneity of behavioral and executive function after moderate and severe pediatric TBI. This work provides the foundation for integrating genomic signatures into future recovery, prognostic, and treatment algorithms for pediatric TBI. It also has implications for future studies attempting to elucidate the association of genetics with outcomes after brain injury more broadly as well. These findings indicate there is great promise to using system biology and genomic approaches to better elucidate how genetic factors influence recovery after TBI. Further exploration of the results indicate that genes associated with axonal guidance biologic pathways are potentially highly involved in long-term cognitive and behavioral outcomes after pediatric TBI.

## TBI genetics and environment study group

Anna Adlam, University of Exeter, Exeter, United Kingdom; Rachel Agbeko, Great North Children’s Hospital, Newcastle upon Tyne, United Kingdom; Alicia Au, Children’s Hospital of Pittsburgh of UPMC, Pittsburgh, PA; Sue Beers, University of Pittsburgh, Pittsburgh, PA; Michael J. Bell, Children’s National Hospital, Washington, DC; Warwick Butt, Murdoch Children’s Research Institute, Royal Children’s Hospital, Melbourne, Australia; Ranjit S. Chima, Cincinnati Children’s Hospital Medical Center, Cincinnati, OH; Robert Clark, University of Pittsburgh, Pittsburgh, PA; Akash Deep, King’s College Hospital, London, United Kingdom; Richard Edwards, Bristol Royal Children’s Hospital for Children, Bristol, United Kingdom; Anthony Fabio, University of Pittsburgh, Pittsburgh, PA; Peter Ferrazzano, University of Wisconsin, Madison, WI; Anthony Figaji, University of Cape Town, Rondebosch, South Africa; Michael Kirkwood, Children’s Hospital Colorado, University of Colorado School of Medicine, Aurora, CO; Michele Kong, Children’s of Alabama, University of Alabama at Birmingham, Birmingham, AL; Kerri LaRovere, Boston Children’s Hospital, Boston, MA; Iain Macintosh, Southampton Children’s Hospital, Southampton General Hospital, Southampton, United Kingdom; Sarah Mahoney, Alder Hey Children’s NHS Foundation Trust, Liverpool, United Kingdom; Lisa J. Martin, Cincinnati Children’s Hospital Medical Center, Cincinnati, OH; Kelly McNally, Nationwide Children’s Hospital, Columbus, OH; Darryl Miles, Children’s Medical Center of Dallas, Dallas, TX; Kevin Morris, Birmingham Women’s and Children’s Hospital, Birmingham, United Kingdom; Nicole O’Brien, Nationwide Children’s Hospital, Columbus, OH; Valentina Pilipenko, Cincinnati Children’s Hospital Medical Center, Cincinnati, OH; Joan Balcells Ramirez, Hospital Vall d’Hebron, Barcelona, Spain; Courtney Robertson, John Hopkins University, Baltimore, MD; Ajit Sarnaik, Children’s Hospital of Michigan, Detroit, MI; Michelle Schober, University of Utah Health, Salt Lake City, UT; Jose Pineda Soto, Washington University, St. Louis, MO and Children’s Hospital of Los Angeles, Los Angeles, CA; Anna Telling, University of Exeter, Exeter, United Kingdom; Hari Thangarajah, Rady Children’s Hospital, San Diego, CA; Neal J. Thomas, Penn State Hershey Children’s Hospital, Hershey, PA; Amery Treble-Barna, University of Pittsburgh, Pittsburgh, PA; Karen Walson, Children’s Healthcare of Atlanta, Atlanta, GA; Alina Nico West, University of Tennessee Health Science Center, Memphis, TN; Anthony Willyerd, Phoenix Children’s Hospital, Phoenix, AZ; Stephen R. Wisniewski, University of Pittsburgh, Pittsburgh, PA; Jerry Zimmerman, Seattle Children’s Hospital, University of Washington School of Medicine, Seattle, WA

## Data Availability

Data for this project have been deposited into the Federal Interagency Traumatic Brain Injury Research Informatics System (FITBIR) as per funding requirement and can be accessed at the following link: https://fitbir.nih.gov/study_profile/351.

## References

[B1] 1000 Genomes Project Consortium Auton A. Brooks L. D. Durbin R. M. Garrison E. P. Kang H. M. (2015). A global reference for human genetic variation. Nature 526 (7571), 68–74. 10.1038/nature15393 26432245 PMC4750478

[B2] Abecasis G. R. Cherny S. S. Cookson W. O. Cardon L. R. (2001). GRR: graphical representation of relationship errors. Bioinformatics 17 (8), 742–743. 10.1093/bioinformatics/17.8.742 11524377

[B3] Beebe D. W. Krivitzky L. Wells C. T. Wade S. L. Taylor H. G. Yeates K. O. (2007). Brief report: parental report of sleep behaviors following moderate or severe pediatric traumatic brain injury. J. Pediatr. Psychol. 32, 845–850. 10.1093/jpepsy/jsm003 17442693 PMC4280793

[B4] Bell M. J. Rosario B. L. Kochanek P. M. Adelson P. D. Morris K. P. Au A. K. (2022). Comparative effectiveness of diversion of cerebrospinal fluid for children with severe traumatic brain injury. JAMA Netw. Open 5 (7), e2220969. 10.1001/jamanetworkopen.2022.20969 35802371 PMC9270700

[B5] Bigos K. L. Hariri A. R. Weinberger D. R. (2016). Neuroimaging genetics: principles and practices. Oxford University Press, 433. Available from: https://books.google.com/books/about/Neuroimaging_Genetics.html?hl=&id=2lrhCgAAQBAJ.

[B6] BRIEF (Behavior Rating Inventory of Executive Function) (2021). Encyclopedia of autism spectrum disorders. 744. 10.1007/978-3-319-91280-6_300262

[B7] Catroppa C. Sood N. T. Morrison E. Kenardy J. Lah S. McKinlay A. (2023). The Australian and New Zealand brain injury lifespan cohort protocol: leveraging common data elements to characterise longitudinal outcome and recovery. BMJ Open 13 (1), e067712. 10.1136/bmjopen-2022-067712 PMC9853218 36657763

[B8] Chapman L. A. Wade S. L. Walz N. C. Taylor H. G. Stancin T. Yeates K. O. (2010). Clinically significant behavior problems during the initial 18 months following early childhood traumatic brain injury. Rehabil. Psychol. 55 (1), 48–57. 10.1037/a0018418 20175634 PMC4137557

[B9] Chen J. Bardes E. E. Aronow B. J. Jegga A. G. (2009). ToppGene Suite for gene list enrichment analysis and candidate gene prioritization. Nucleic Acids Res. 37, W305–W311. 10.1093/nar/gkp427 19465376 PMC2703978

[B10] Chen J. Xu H. Aronow B. J. Jegga A. G. (2007). Improved human disease candidate gene prioritization using mouse phenotype. BMC Bioinforma. 8, 392. 10.1186/1471-2105-8-392 PMC2194797 17939863

[B11] Chevignard M. P. Servant V. Mariller A. Abada G. Pradat-Diehl P. Laurent-Vannier A. (2009). Assessment of executive functioning in children after TBI with a naturalistic open-ended task: a pilot study. Dev. Neurorehabilitation 12, 76–91. 10.1080/17518420902777019 19340660

[B12] Cohen J. (1988). Statistical power analysis for the behavioral sciences. 2nd Edition. New York: Routledge, 567. Available from: https://api.taylorfrancis.com/content/books/mono/download?identifierName=doi&identifierValue=10.4324/9780203771587&type=googlepdf (Accessed April 26, 2023).

[B13] Conklin H. M. Salorio C. F. Slomine B. S. (2008). Working memory performance following paediatric traumatic brain injury. Brain Inj. 22, 847–857. 10.1080/02699050802403565 18850343

[B14] Cortes D. Pera M. F. (2021). The genetic basis of inter-individual variation in recovery from traumatic brain injury. NPJ Regen. Med. 6 (1), 5. 10.1038/s41536-020-00114-y 33479258 PMC7820607

[B15] Dixson L. Tost H. Meyer-Lindenberg A. (2018). “Imaging genetics,”in Psychiatric genetics, 107–123. 10.1093/med/9780190221973.003.0008

[B16] Donders J. DenBraber D. Vos L. (2010). Construct and criterion validity of the Behaviour Rating Inventory of Executive Function (BRIEF) in children referred for neuropsychological assessment after paediatric traumatic brain injury. J. Neuropsychol. 4 (2), 197–209. 10.1348/174866409X478970 19930791

[B17] Ewing-Cobbs L. Barnes M. Fletcher J. M. Levin H. S. Swank P. R. Song J. (2004). Modeling of longitudinal academic achievement scores after pediatric traumatic brain injury. Dev. Neuropsychol. 25 (1-2), 107–133. 10.1080/87565641.2004.9651924 14984331

[B18] Fan J. B. Oliphant A. Shen R. Kermani B. G. Garcia F. Gunderson K. L. (2003). Highly parallel SNP genotyping. Cold Spring Harb. Symp. Quant. Biol. 68, 69–78. 10.1101/sqb.2003.68.69 15338605

[B19] Fitzgerald M. Ponsford J. Hill R. Rushworth N. Kendall E. Armstrong E. (2023). The Australian Traumatic Brain Injury Initiative: single data dictionary to predict outcome for people with moderate-severe traumatic brain injury. J. Neurotrauma. 10.1089/neu.2023.0467 38117144

[B20] Fletcher J. M. Ewing-Cobbs L. Miner M. E. Levin H. S. Eisenberg H. M. (1990). Behavioral changes after closed head injury in children. J. Consult. Clin. Psychol. 58, 93–98. 10.1037/0022-006x.58.1.93 2319050

[B21] Galwey N. W. (2023). A Q-Q plot aids interpretation of the false discovery rate. Biom J. 65 (1), e2100309. 10.1002/bimj.202100309 35839474

[B22] Gaysina D. (2022). “Behavioral genetics,” in The wiley‐blackwell handbook of childhood social development, 43–60. 10.1002/9781119679028.ch2

[B23] Gioia G. Isquith P. (2004). Ecological assessment of executive function in traumatic brain injury. Dev. Neuropsychol. 25, 135–158. 10.1080/87565641.2004.9651925 14984332

[B24] Gioia G. A. Isquith P. K. Guy S. C. Kenworthy L. (2000). TEST REVIEW behavior rating inventory of executive function. Child. Neuropsychol. 6, 235–238. 10.1076/chin.6.3.235.3152 11419452

[B25] Gioia G. A. Isquith P. K. Kenworthy L. Barton R. M. (2002). Profiles of everyday executive function in acquired and developmental disorders. Child. Neuropsychol. 8 (2), 121–137. 10.1076/chin.8.2.121.8727 12638065

[B26] Goddard M. E. Kemper K. E. MacLeod I. M. Chamberlain A. J. Hayes B. J. (2016). Genetics of complex traits: prediction of phenotype, identification of causal polymorphisms and genetic architecture. Proc. Biol. Sci. 283 (1835), 20160569. 10.1098/rspb.2016.0569 27440663 PMC4971198

[B27] Greene C. M. Braet W. Johnson K. A. Bellgrove M. A. (2008). Imaging the genetics of executive function. Biol. Psychol. 79, 30–42. 10.1016/j.biopsycho.2007.11.009 18178303

[B28] Hillmer R. A. (2015). Systems biology for biologists. PLoS Pathog. 11 (5), e1004786. 10.1371/journal.ppat.1004786 25973920 PMC4431723

[B29] Karunanayaka P. R. Holland S. K. Yuan W. Altaye M. Jones B. V. Michaud L. J. (2007). Neural substrate differences in language networks and associated language-related behavioral impairments in children with TBI: a preliminary fMRI investigation. NeuroRehabilitation 22 (5), 355–369. 10.3233/nre-2007-22503 18162699 PMC4280792

[B30] Kenzie E. S. Parks E. L. Bigler E. D. Lim M. M. Chesnutt J. C. Wakeland W. (2017). Concussion as a multi-scale complex system: an interdisciplinary synthesis of current knowledge. Front. Neurol. 8, 513. 10.3389/fneur.2017.00513 29033888 PMC5626937

[B31] Kenzie E. S. Parks E. L. Carney N. Wakeland W. (2022). System dynamics modeling for traumatic brain injury: mini-review of applications. Front. Bioeng. Biotechnol. 10, 854358. 10.3389/fbioe.2022.854358 36032727 PMC9411712

[B32] Kochanek P. M. Adelson P. D. Rosario B. L. Hutchison J. Miller Ferguson N. Ferrazzano P. (2022). Comparison of intracranial pressure measurements before and after hypertonic saline or mannitol treatment in children with severe traumatic brain injury. JAMA Netw. Open 5 (3), e220891. 10.1001/jamanetworkopen.2022.0891 35267036 PMC8914575

[B33] Kurowski B. G. Backeljauw B. Zang H. Zhang N. Martin L. J. Pilipenko V. (2016). Influence of catechol-O-methyltransferase on executive functioning longitudinally after early childhood traumatic brain injury: preliminary findings. J. Head. Trauma Rehabil. 31 (3), E1–E9. 10.1097/HTR.0000000000000162 PMC4724555 26394291

[B34] Kurowski B. G. Haarbauer-Krupa J. Giza C. C. (2022). When traumatic brain injuries in children become chronic Health conditions. J. Head. Trauma Rehabil. 38, 348–350. 10.1097/HTR.0000000000000842 36584980 PMC10310882

[B35] Kurowski B. G. Treble-Barna A. Pilipenko V. Wade S. L. Yeates K. O. Taylor H. G. (2019). Genetic influences on behavioral outcomes after childhood TBI: a novel systems biology-informed approach. Front. Genet. 10, 481. 10.3389/fgene.2019.00481 31191606 PMC6540783

[B36] Kurowski B. G. Treble-Barna A. Pitzer A. J. Wade S. L. Martin L. J. Chima R. S. (2017a). Applying systems biology methodology to identify genetic factors possibly associated with recovery after traumatic brain injury. J. Neurotrauma 34 (14), 2280–2290. 10.1089/neu.2016.4856 28301983 PMC5510694

[B37] Kurowski B. G. Treble-Barna A. Zang H. Zhang N. Martin L. J. Yeates K. O. (2017b). Catechol-O-Methyltransferase genotypes and parenting influence on long-term executive functioning after moderate to severe early childhood traumatic brain injury: an exploratory study. J. Head. Trauma Rehabil. 32 (6), 404–412. 10.1097/HTR.0000000000000281 28060209 PMC5498281

[B38] Marion C. M. Radomski K. L. Cramer N. P. Galdzicki Z. Armstrong R. C. (2018). Experimental traumatic brain injury identifies distinct early and late phase axonal conduction deficits of white matter pathophysiology, and reveals intervening recovery. J. Neurosci. 38 (41), 8723–8736. 10.1523/JNEUROSCI.0819-18.2018 30143572 PMC6181309

[B39] McCauley S. R. Wilde E. A. Anderson V. A. Bedell G. Beers S. R. Campbell T. F. (2012). Recommendations for the use of common outcome measures in pediatric traumatic brain injury research. J. Neurotrauma 29 (4), 678–705. 10.1089/neu.2011.1838 21644810 PMC3289848

[B40] Merkley T. L. Bigler E. D. Wilde E. A. McCauley S. R. Hunter J. V. Levin H. S. (2008). Diffuse changes in cortical thickness in pediatric moderate-to-severe traumatic brain injury. J. Neurotrauma 25 (11), 1343–1345. 10.1089/neu.2008.0615 19061377 PMC2747789

[B41] Muscara F. Catroppa C. Anderson V. (2008a). Social problem-solving skills as a mediator between executive function and long-term social outcome following paediatric traumatic brain injury. J. Neuropsychol. 2 (2), 445–461. 10.1348/174866407x250820 19824165

[B42] Muscara F. Catroppa C. Anderson V. (2008b). The impact of injury severity on executive function 7-10 years following pediatric traumatic brain injury. Dev. Neuropsychol. 33 (5), 623–636. 10.1080/87565640802171162 18788014

[B43] North B. V. Curtis D. Sham P. C. (2002). A note on the calculation of empirical P values from Monte Carlo procedures. Am. J. Hum. Genet. 71 (2), 439–441. 10.1086/341527 12111669 PMC379178

[B44] Nunes A. P. Oliveira I. O. Santos B. R. Millech C. Silva L. P. González D. A. (2012). Quality of DNA extracted from saliva samples collected with the Oragene^TM^ DNA self-collection kit. BMC Med. Res. Methodol. 12, 65. 10.1186/1471-2288-12-65 22559914 PMC3422993

[B45] Patterson N. Price A. L. Reich D. (2006). Population structure and eigenanalysis. PLoS Genet. 2 (12), e190. 10.1371/journal.pgen.0020190 17194218 PMC1713260

[B46] Petranovich C. L. Smith-Paine J. Wade S. L. Yeates K. O. Taylor H. G. Stancin T. (2020). From early childhood to adolescence: lessons about traumatic brain injury from the Ohio head injury outcomes study. J. Head. Trauma Rehabil. 35 (3), 226–239. 10.1097/HTR.0000000000000555 31996606 PMC7210058

[B47] Power T. Catroppa C. Coleman L. Ditchfield M. Anderson V. (2007). Do lesion site and severity predict deficits in attentional control after preschool traumatic brain injury (TBI)? Brain Inj. Brain Inj. 21 (3), 279–292. 10.1080/02699050701253095 17453756

[B48] Purcell S. Neale B. Todd-Brown K. Thomas L. Ferreira M. A. R. Bender D. (2007). PLINK: a tool set for whole-genome association and population-based linkage analyses. Am. J. Hum. Genet. 81 (3), 559–575. 10.1086/519795 17701901 PMC1950838

[B49] Reddi S. Thakker-Varia S. Alder J. Giarratana A. O. (2022). Status of precision medicine approaches to traumatic brain injury. Neural Regen. Res. 17 (10), 2166–2171. 10.4103/1673-5374.335824 35259824 PMC9083178

[B50] Rempe G. Petranovich C. Narad M. E. Yeates K. O. Taylor H. G. Stancin T. (2022). Trajectories of executive functions after early childhood traumatic brain injury: teacher ratings in the initial 81 Months postinjury. J. Head. Trauma Rehabil. 38, E203–E211. 10.1097/HTR.0000000000000833 36730995 PMC10102250

[B51] Schwartz L. Taylor H. G. Drotar D. Yeates K. O. Wade S. L. Stancin T. (2003). Long-term behavior problems following pediatric traumatic brain injury: prevalence, predictors, and correlates. J. Pediatr. Psychol. 28 (4), 251–263. 10.1093/jpepsy/jsg013 12730282

[B52] Sesma H. W. Slomine B. S. Ding R. McCarthy M. L. Children’s Health After Trauma (CHAT) Study Group (2008). Executive functioning in the first year after pediatric traumatic brain injury. Pediatrics 121 (6), e1686–e1695. 10.1542/peds.2007-2461 18519472

[B53] SNP Genotyping and Copy Number Analysis (2023). SNP genotyping and Copy number analysis. Available from: https://www.illumina.com/content/dam/illumina-marketing/documents/products/guides/2009_product_guide_snp_genotyping_cnv.pdf (Accessed May 1, 2023).

[B54] Stancin T. Drotar D. Taylor H. G. Yeates K. O. Wade S. L. Minich N. M. (2002). Health-related quality of life of children and adolescents after traumatic brain injury. Pediatrics 109 (2), E34. 10.1542/peds.109.2.e34 11826244

[B55] Taylor H. G. Yeates K. O. Wade S. L. Drotar D. Stancin T. Burant C. (2001). Bidirectional child-family influences on outcomes of traumatic brain injury in children. J. Int. Neuropsychol. Soc. 7 (6), 755–767. 10.1017/s1355617701766118 11575597

[B56] Taylor H. G. Yeates K. O. Wade S. L. Drotar D. Stancin T. Minich N. (2002). A prospective study of short- and long-term outcomes after traumatic brain injury in children: behavior and achievement. Neuropsychology 16 (1), 15–27. 10.1037//0894-4105.16.1.15 11853353

[B57] Teasdale G. Jennett B. (1974). Assessment of coma and impaired consciousness. A practical scale. Lancet 2 (7872), 81–84. 10.1016/s0140-6736(74)91639-0 4136544

[B58] TOPMed Imputation ServerFree Next-Generation Genotype Imputation Service (2024). TOPMed imputation ServerFree next-generation genotype imputation service. Website. Available from: https://imputation.biodatacatalyst.nhlbi.nih.gov/.

[B59] Treble-Barna A. Pilipenko V. Wade S. L. Jegga A. G. Yeates K. O. Taylor H. G. (2020). Cumulative influence of inflammatory response genetic variation on long-term neurobehavioral outcomes after pediatric traumatic brain injury relative to orthopedic injury: an exploratory polygenic risk score. J. Neurotrauma 37 (13), 1491–1503. 10.1089/neu.2019.6866 32024452 PMC7307697

[B60] Treble-Barna A. Wade S. L. Martin L. J. Pilipenko V. Yeates K. O. Taylor H. G. (2017). Influence of dopamine-related genes on neurobehavioral recovery after traumatic brain injury during early childhood. J. Neurotrauma 34 (11), 1919–1931. 10.1089/neu.2016.4840 28323555 PMC5455258

[B61] Treble-Barna A. Wade S. L. Pilipenko V. Martin L. J. Yeates K. O. Taylor H. G. (2022). Brain-derived neurotrophic factor Val66Met and behavioral adjustment after early childhood traumatic brain injury. J. Neurotrauma 39 (1-2), 114–121. 10.1089/neu.2020.7466 33605167 PMC8785712

[B62] Treble-Barna A. Wade S. L. Pilipenko V. Martin L. J. Yeates K. O. Taylor H. G. (2023). Brain-derived neurotrophic factor Val66Met and neuropsychological functioning after early childhood traumatic brain injury. J. Int. Neuropsychol. Soc. 29 (3), 246–256. 10.1017/S1355617722000194 35465864 PMC9592678

[B63] Turner S. Armstrong L. L. Bradford Y. Carlson C. S. Crawford D. C. Crenshaw A. T. (2011). Quality control procedures for genome-wide association studies. Curr. Protoc. Hum. Genet. 1. 10.1002/0471142905.hg0119s68 PMC3066182 21234875

[B64] Vriezen E. R. Pigott S. E. (2002). The relationship between parental report on the BRIEF and performance-based measures of executive function in children with moderate to severe traumatic brain injury. Child. Neuropsychol. 8 (4), 296–303. 10.1076/chin.8.4.296.13505 12759826

[B65] Wade S. L. Taylor H. G. Drotar D. Stancin T. Yeates K. O. Minich N. M. (2002). A prospective study of long-term caregiver and family adaptation following brain injury in children. J. Head. Trauma Rehabil. 17 (2), 96–111. 10.1097/00001199-200204000-00003 11909509

[B66] Walz N. C. Cecil K. M. Wade S. L. Michaud L. J. (2008). Late proton magnetic resonance spectroscopy following traumatic brain injury during early childhood: relationship with neurobehavioral outcomes. J. Neurotrauma 25 (2), 94–103. 10.1089/neu.2007.0362 18260792 PMC4278195

[B67] Wang K. Li M. Hakonarson H. (2010). ANNOVAR: functional annotation of genetic variants from high-throughput sequencing data. Nucleic Acids Res. 38 (16), e164. 10.1093/nar/gkq603 20601685 PMC2938201

[B68] Wozniak J. R. Krach L. Ward E. Mueller B. A. Muetzel R. Schnoebelen S. (2007). Neurocognitive and neuroimaging correlates of pediatric traumatic brain injury: a diffusion tensor imaging (DTI) study. Arch. Clin. Neuropsychol. 22 (5), 555–568. 10.1016/j.acn.2007.03.004 17446039 PMC2887608

[B69] Yang L. Wang P. Chen J. (2024). 2dGBH: two-dimensional group Benjamini-Hochberg procedure for false discovery rate control in Two-Way multiple testing of genomic data. Bioinformatics 40, btae035. 10.1093/bioinformatics/btae035 38244568 PMC10873908

[B70] Yeates K. O. Taylor H. G. Wade S. L. Drotar D. Stancin T. Minich N. (2002). A prospective study of short- and long-term neuropsychological outcomes after traumatic brain injury in children. Neuropsychology 16 (4), 514–523. 10.1037//0894-4105.16.4.514 12382990

[B71] Yu T. S. Washington P. M. Kernie S. G. (2016). Injury-induced neurogenesis: mechanisms and relevance. Neuroscientist 22 (1), 61–71. 10.1177/1073858414563616 25520428

