# Supplementary material for: Elucidating a genomic signature associated with behavioral and executive function after moderate to severe pediatric TBI: a systems biology informed approach
Source: Front Syst Biol. 2024 Apr 25;4:1293265. doi: 10.3389/fsysb.2024.1293265 (PMC12342039; doi:10.3389/fsysb.2024.1293265)
Supplement: Supplementary file 2 [file Table1.DOCX]

Supplementary Table 1: Participant characteristics by injury type and cohort

| **Characteristic** | **Overall**,  N = 196*^1^* | **OHIO-OI**,  N = 61*^1^* | **OHIO-Moderate**, N = 49*^1^* | **OHIO-Severe**,  N = 14*^1^* | **ADAPT-Severe**,  N = 72*^1^* |
| --- | --- | --- | --- | --- | --- |
| Sex |  |  |  |  |  |
| Female | 103 (53%) | 30 (49%) | 23 (47%) | 6 (43%) | 44 (61%) |
| Male | 93 (47%) | 31 (51%) | 26 (53%) | 8 (57%) | 28 (39%) |
| GCS | 7 (4, 13) | NA | 14 (13, 15) | 3 (3, 3) | 6 (3, 7) |
| Missing data | 1 | NA | 0 | 0 | 1 |
| Age at injury, years | 5.7 (4.3, 7.2) | 5.2 (4.2, 6.0) | 5.2 (4.1, 6.1) | 5.1 (4.6, 5.9) | 11.9 (4.8, 14.2) |
| Time since injury, days | 396 (377, 424) | 404 (389, 423) | 423 (398, 434) | 408 (393, 433) | 376 (365, 391) |
| Ancestral populations |  |  |  |  |  |
| Ad Mixed American | 11 (5.6%) | 1 (1.6%) | 2 (4.1%) | 0 (0%) | 8 (11%) |
| African | 34 (17%) | 9 (15%) | 11 (22%) | 4 (29%) | 10 (14%) |
| East Asian | 2 (1.0%) | 0 (0%) | 0 (0%) | 0 (0%) | 2 (2.8%) |
| European | 146 (74%) | 51 (84%) | 36 (73%) | 10 (71%) | 49 (68%) |
| South Asian | 3 (1.5%) | 0 (0%) | 0 (0%) | 0 (0%) | 3 (4.2%) |
| Ethnicity |  |  |  |  |  |
| Hispanic or Latino | 6 (3.1%) | 3 (4.9%) | 2 (4.1%) | 0 (0%) | 1 (1.4%) |
| Not Hispanic or Latino | 165 (84%) | 58 (95%) | 47 (96%) | 14 (100%) | 46 (64%) |
| Data not available | 25 (13%) | 0 (0%) | 0 (0%) | 0 (0%) | 25 (35%) |
| CBCL | 50 (41, 59) | 41 (38, 50) | 52 (42, 57) | 58 (53, 65) | 56 (47, 64) |
| Missing data | 4 | 0 | 0 | 0 | 4 |
| BRIEF | 53 (43, 63) | 45 (41, 52) | 55 (42, 61) | 60 (56, 68) | 60 (50, 70) |
| Missing data | 3 | 0 | 0 | 0 | 3 |
| *^1^* n (%); Median (IQR) | | | | | |
